# Keyword index

**DOI:** 10.1038/sj.bjc.6690640

**Published:** 1999-08


					ABC transporters 1342
1
-Acid glycoprotein 1738
Acid-base balance 1005
Acidic stress 1892
Actinic keratosis 1087
Acute lymphoblastic leukaemia 909
ADCC 331
Adenocarcinoma 51, 70, 1652
Adenocarcinoma oesophagus 1652
Adhesion 1867
Adjuvant chemotherapy 1763, 1767
Adrenal enlargement 1815
Aflatoxin 59
ALA 185, 676, 1525
ALA derivatives 1525
ALA esters 1525
ALA hexylester 185
-ALA 998
Alcohol 1476
Alkylating agents 338
Allelic imbalance 850
Allergies 1830
Alternative splice 1185
Aluminium phthalocyanine 1533
Aminolaevulinic acid 676
5-Aminolaevulinic acid 185, 1525
5-Aminolaevulinic acid derivatives 1525
5-Aminolaevulinic acid esters 1525
5-Aminolaevulinic acid hexylester 185
-Aminolaevulinic acid 998
Anal carcinoma 1588
Anaplastic carcinoma 650
Androgen independence 546
Androstanediol glucuronide 930
Angiogenesis 17, 104, 142, 733, 892, 898,
1697
Animal models 149
Antagonist G 1026
Antenatal environmental studies 1844
Anti-emetics 412
Anti-microtubule agents 1162
Anti-p53 antibodies 1987
Anti-tumour promoters 110
Anti-tumour antibody-enzyme conjugates
1747
Antibody responses 219
Anticancer agents 991
Antioxidants 25
Antisense oligonucleotides 1020
Antisense RNA 1115
Anxiety 1770
APE 940
APEX 940
Apoptosis 1, 51, 371, 546, 705, 711, 756,
1026, 1035, 1062, 1069, 1075, 1197, 1350,
1550, 1558, 1689, 1754, 1892
Apurinic sites 940
ara-C 1542
Arsenic-related skin cancers 1080
Arteriolar diameter 117
Assessments 792
Assessments of response 221
Ataxia telangiectasia 1979
Ataxia telangiectasia heterozygosis 1042
ATX-S10 133
Audits 444, 563
Autocrine loop 657
Autologous peripheral blood transplantation
229
Axillary lymph nodes 1974
AZT 87
Bax 1892
BCL 10 1491, 1565, 1569, 1571, 1575
bcl-2 1658
Bcl-2 proteins 1075
213
Bi 175
Bicarbonate 1005
Bioavailability 1380
Biological markers 1281
Biricodar 1190
Biweekly schedule 1052
Bladder 273, 904
Bladder cancer 489, 1312
Bladder cancer, human 185
Bladder neoplasms 1175
Blindness 1459
Blood-brain barrier 964
Blood donors 495
Blood flow 117
Blood platelets 892
Body mass indices 1470
Bone marrow 87
Bone metastases 221, 1265
Bone resorption markers 221
BPD-MA 344, 946
Brain tumours 383, 756
Breaking bad news 792
Breast cancer 221, 229, 262, 286, 352,
379, 419, 519, 536, 563, 579, 609, 821,
874, 879, 898, 1092, 1150, 1262, 1265,
1453, 1470, 1838, 1905, 1968, 1974,
1979, 1982, 2019
Breast cancer, early onset 167, 843
Breast cancer risk 1042
Breast-conserving surgery 167
Breast neoplasms 1459, 1763, 1852
Bronchus 744, 1435
Burkitt's lymphoma 670
Butter 614
Bystander effects 1726
CA125 1644
Cadherin 322
Cadherin-catenin complex 1046
Calcium 1107
Calnexin 639
Calreticulin 639
Cancer 87, 215, 269, 273, 279, 495, 792,
898, 1092, 1440, 1770, 1844
Cancer cachexia 1734
Cancer consultation 427
Cancer gene therapy 1726
Cancer predisposition 1979
Cancer registry 1826 (SC)
Cancer treatment 1275
Canine mammary tumours 1359
Carbogen 117
Carbon dioxide 117
Carcinogens 855
Carcinoma cells 1689
Cartilage 1359
Case-control studies 585, 621, 1098,
1483, 1859
Caspases 371, 1892
Caspase-3 711
Catenin 322
-Catenin 477
-Catenin 1046
Cathepsin-D 167
CD30 1214
CD44 918
CD95 1689
Cdc25A 1144
cdk inhibitors 1144, 1427
cdkl 670
CDKN2 9
CDKN2A genes 1920
Cell adhesion 322
Cell cycles 971, 1905
Cell cycle arrest 1144
Cell invasion 733
Cell motility factors 1708
Cell polarity 1162
Cell proliferation 2001
Cellular adhesion 946
Ceramide 693
c-erbB2/neu 519
Cerebral PNETs 1322
Cervical cancer 403, 458, 827, 1306, 1387,
1518, 1828 (SC), 1958, 1599, 2008
Cervical intra-epithelial neoplasia 621
Characterization 149
Checklists 563
Chemoprotection 629
Chemotherapy 142, 262, 269, 396, 403, 438,
756, 775, 801, 815, 964, 1005, 1035, 1058,
1332, 1577, 1588, 1797, 1962
Chemosensitizers 1190
Chernobyl 1461
Childhood 1844
Childhood Hodgkin's disease 808
Childhood leukaemia 585
Chinese 1828 (SC)
Cholangiocellular carcinoma 1069
Cholecystectomy 1830
Chondroitin sulphate 1359
Chorionic cancer, human 1577, 1582
Chromosome 9 489
Chromosome 10 904
Keyword index
Key to abbreviations:
(SC) Short communications
2048
British Journal of Cancer (1999) 80(12), 2048-2052
(c) 1999 Cancer Research Campaign
Article no. bjoc.1999.0640
Keyword index 2049
British Journal of Cancer (1999) 80(12), 2048-2052
(c) 1999 Cancer Research Campaign
Chromosome 11q23 879
Chromosome 17p13.3 821
Chromosomal aberrations 2034
Chromosomal gains 526
Chromosomal instability 862, 1312
Chromosomal losses 526
Chronic hepatitis B virus carriers 598
Cigarette smoking 598
Circulating cancer cells 883
Cisplatin 699, 815, 954, 981, 1052, 1245,
1332, 1665, 1912, 1962
Cisplatin chemotherapy 1387
Cisplatin-based chemotherapy 1392
CK20 448
Classification 834
Clinical outcomes 1289
Clinical trials 236
Clonality 909
CMF 1763
cMOAT 1342
Co-culture 1137
Coagulopathy 279
Cohort studies 609, 1107, 1476
Collagen 315
32
P-Colloid 1955
Colo-26 tumours 1533
Colon 38 716
Colon cancer 127, 364, 699, 1132, 1373,
1506, 1718, 1884
Colon cancer cell lines 1012
Colorectal adenoma 194
Colorectal cancers 194, 786, 922, 1046,
1062, 1569, 1635, 1867, 1995
Colorectal tumours 526, 914
Communication 427
Communication skills 792
Comparative genomic hybridization 862,
1322, 2034
Complement 1820
Conservative surgery 403
Costs 215, 815
Counselling 1770
CP94 1525
CpG islands 1262
CPT-11 364
Cyclin 256
Cyclin A 2001
Cyclin D1 79, 705, 1175, 1281, 1289, 2001
Cyclin-dependent kinases 256, 991
Cyclophosphamide 87
CYP1A1 1838
CYP1A1 1445
CYP2D6 1445
Cytochrome P450 1A1 598
Cytokines 504, 579
Cytological screening 1306
Cytoskeleton 1867
Cytosol 286
Cytotoxicity 1223, 1786
Dairy 1107
Daudi cells 1542
DCC 51
DEAD box protein/RNA helicase 914
Deletion mapping 489
Depression 1770
Dermal fibroblasts, human 1137
Dexamethasone 1815
5-DFUR 1726
Diabetes mellitus 1830
Diets 614
Differentiating agents 1252
Differentiation 1, 96, 1123, 1156, 1608
Diffuse large B-cell lymphoma 1427
Dipyridamole analogues 1738
Direct CGH 526
DMXAA 716
DNA adducts 981
DNA binding 991
DNA damage 981
DNA fragmentation 711
DNA interstrand cross-links 1245
DNA methylation 1262, 1982
DNA mismatch repairs 11, 699
DNA polymerase- 971
Docetaxel 438, 1792
Dopamine D2
antagonists 412
Dose reconstruction 1461
Doxorubicin 711, 1005, 1204
Drug deliveries 964, 1718
Drug resistance 1, 1020, 1912
DT-diaphorase 1223
Early breast cancer 167
Early-stage central-type of squamous cell
carcinoma 1435
EBV-EA inhibition 110
E-Cadherin 273, 1281, 1366, 1652
ECF 269
ECM 1867
EGF-receptors 657
Eicosapentaenoic acid (EPA) 1231
Electrical fields 1204
Electrochemotherapy 1204
ELISA 286, 495
Embryonal rhabdomyosarcoma 403
EMT-6 tumours 1533
Endometrial cancer 458, 699
Endoscopic features 1435
Endotoxins 716
Enhancers 1935
Enzyme inducers 1223
Epidemiology 834, 1440, 1459, 1483, 1844
Epidermal growth factor receptors 1012,
1987
Epirubicin 1797
Epithelial cells 1550
Epoetin 396
Epstein-Barr virus 604, 670
ER 579
E-Selectin 1169
Exercise 1852
Experimental models 1373
Extracellular matrix 1087, 1359
Extracellular matrix proteins 946
Faecal coprostanol 1132
Familial melanoma 295
Familial cancer 1830
Family studies 1042
FAA 1905
FAMTX 269
Fas 371
FDG PET 513
Fertility 249
Fertility aspects 801
FFA 513
FHIT 73
Fibroblasts 315, 676
Fibronectin 1359
Fine-needle aspiration biopsies 73, 519, 1672
FISH 519
Flavone acetic acid 1905
Flow cytometry 874, 1062
Fluorescence 185
5-Fluorodeoxyuridine 1718
5-Fluorouracil 786, 954, 1726
5-Fluorouracil cytotoxicity 1738
FNA biopsies 73, 519, 1672
Focal nodular hyperplasia 73
Follow-up studies 1826 (SC)
Food 1380
Fos 1512
5-FU 786, 954, 1726
G1 checkpoint 79
G2/M cell cycle arrest 1905
G361 melanoma 1734
GAGE-1 73
GAGE-2 73
Gains 526
Gastric adenocarcinoma 483
Gastric cancer 206, 269, 556, 1271, 1630
Gastric cancer cells 1144
Gastric carcinoma 322, 834
G-CSF 229
GDNF 383
GDNFR 383
Gelatinase 733
Gelatinase B 504
Gemcitabine 981, 1797
Gene expression 1123
Gene therapy 1420
Gene up-regulation 1518
Genetic damage 2008
Genetic instability 453
Genetic polymorphism 1838
Genetic susceptibility 855
Genistein 1150, 1550, 1682
Germ cell malignancy 1392
Germ cell mutations 909
Germ cell neoplasia 569
Germany 585, 1440
Gestational age 609
GFR-1 383
Glial fibrillary acidic protein 1322
Glioma cells 756
Gliomas 104, 142, 964, 1558
Glucocorticoids 96
Glucose metabolism 513
Glucose utilization 1231
Glutathione 629
Glutathione-S-transferase M1 598
Glycolysis 1035
GnRH 352
Gonadotrophins 1577, 1582
G-proteins 1026
Granisetron 412
Granulosa cells 70
GSH peroxidase 32
GSH-S-transferase P1-isoenzymes 32
GSSG-reductase 32
GSTM1 1445
GTBP 1665
HAD scale 766
Haem oxygenase-1 1945
Head and neck cancer 766, 1400
Head and neck squamous cell cancers 1565
HeLa cell tumours 133
2050 Keyword index
British Journal of Cancer (1999) 80(12), 2048-2052 (c) 1999 Cancer Research Campaign
Hepatitis B virus 59
Hepatitis C virus 1303
Hepatocellular carcinomas 59, 73, 468, 598,
850, 1820, 2034
Hepatomas 705, 1197
Hepatoma cells, multidrug resistant 1197
Hepatoma cells, rat 711
Herpesvirus-type 8, human 67
H-Cadherin 556
HGF 1708
High dost MTX 1781
Histology 1859
Histone acetylation 1252
Histopathological findings 1435
HL-60 cells 1156
HLA-C 639
HLA-DPB1 1405
hMLH1 699
hMLH1 inactivation 11
hMSH2 699, 1665
hMSH2 mutations 11
Hodgkin's disease 604, 1405
Hodgkin's lymphoma 1214
Hormones 1058
Hormone resistance 1982
HRT 1453
HT-29 cells 360
hTERT 1156
Human herpes virus-6 1103
HVR 1405
Hybridization in situ 206, 1087
Hyperbaric oxygenation 236
Hyperthermia 1387
Hypoxia 104, 1245, 1518, 1697, 1875
Iaminin receptors 1115
ICAM-1 954, 1494
ICG 360
Immune system 585
Immunocytochemistry 519
Immunohistochemistry 167, 458, 922, 1062,
1075, 1920
Immunoliposomes 1718
Immunotherapy 331, 352, 407, 775, 1767
In situ hybridization 206, 1087
In utero studies 1092
In vivo animal models 1420
In vivo elimination 1582
Incidences 1440
Infections 585, 1483
Influenza 1483
Influenza vaccine 219
INK4A 295
INK4D 295
Integrins 1867
6-Integrin 1927
Intensive chemotherapy 1595
Interferons 705
Interferon- 1350, 1767, 1781
Interferon- 786
Interferon- 127, 1236, 1781
Interferon- receptors 127
Interleukin-1 1506
Interleukin-2 407
Interleukin-6 67, 407, 579, 892, 1617
Interleukin-15 1420
Interventions 242
Intestine 1123, 1550
Intracavital therapies 775
Invasion 1412, 2025
Iodine-131 1461
Ionizing radiation 940, 1689
IRF-1 1236
Irinotecan 364
Iron chelators 1525
Irradiation 801
Isochromosome 17q 1322
Isoflavones 1550
Isolated limb perfusion 161
IUDR 1635
JNK 1026
Ki-4 1214
Ki-67 387, 579, 1809
Ki-67 index 1289
Kinases, cyclin-dependent 256, 991
Knockout 1884
K-Ras oncogene 1884
Labelling indices 1635
LAK cells 1494
Laminin 1898, 1927
Lectin 1754
Letters to other doctors 427
Leucopenia 1763
Leukaemia 909, 1103, 1483, 1844
Leukaemia cells 1898
LEXF 855
Li-Fraumeni syndrome 9
Ligand and proliferation 1012
Light dosimetry 744
Lipid diffusion 1204
Lipid peroxidation 360, 1204
Lipophilic derivatives 1542
Liposomes 1542
Liver 1820
Liver tissues 364
Liver metastases 1373
LKB1/STK11 70
Local controls 387
Local recurrence 167
LOH 44, 59, 468, 489, 556, 821, 879, 904,
1652, 1920, 2008
LOH analysis 843
Loss of heterozygosity 44, 59, 468, 489, 556,
821, 879, 904, 1652, 1920, 2008
Loss of heterozygosity analysis 843
Low density lipoproteins 1542
Luminometric immunoassays 536
Lung adenocarcinoma, human 1169
Lung cancer 96, 215, 219, 453, 1020, 1115,
1565, 1623, 1792, 1987
Lymph node metastases 309, 483, 1366, 2019
Lymphocytes 874, 1542
Lymphocytosis 407
Lymphoma 331, 513, 1476
Lymphoproliferative disorders 1303
Magnetic resonance spectroscopy 1005, 1035
Malformations 1092
Malignant effusion 775
Malignant endocrine pancreatic tumours
1259
Malignant gliomas 236
Malignant melanomas 67
Malignant transformation 676
Mammalian cell cycles 1875
Mammary tumorigenesis 1682
MAPK 1412
Marker expressions 1608
Material deprivation 604
Matrix metalloprotease-1 935
Matrix metalloproteinases 17, 1087
Matrix metalloproteinases, tissue inhibitors of
17
mdm2 38, 1185
MDM2 73
mdr1a 1123
mdr1b 1123
Meat 1107
Medical record linkage 1459
Medulloblastoma 1322
MelanA/MART-1 883
Melanoma 504, 639, 883, 1252, 1494, 1672,
1697, 1767, 2025
Melatonin 1058, 1459
Melphalan 161
Membrane cofactor proteins 1820
Membrane type-1 matrix metalloproteinase
1137
MEN1 44
Menin transcripts 44
Mesothelioma 25
Met 650
Meta-analysis 1770
Metastasis 38, 167, 301, 448, 1169, 1697,
1867, 1927, 1962
Methylation 458
Methylcytosine 1312
Metopimazine 412
MIB1 1427
2
-Microglobulin 639
Micronucleus assays 1599
Microsatellites 468, 556
Microsatellite instability 1803
Microtubule-associated proteins 1162
Microvascular corrosion castings 724
Midgut carcinoids 1259
Minimal breast cancer 1608
Minor groove binders 338, 991
Mismatch repairs 338
Mitochondria 25
Mitomycin 1962
Mitomycin C 1223
MMP 733
MMP-2 315
MMP-9 1366, 1412
Molecular detection 1803
Molecular markers 1281, 2019
Monoclonal antibodies 569, 1718
Monocyte tissue factor 279
Monosomy 22 1322
Motility 127
Mouse 149, 1115
mRNA 206
mRNA stability 657
MRP 1342
MRS 1005, 1035
mTHPC 744
MUC1 mucin 301
Multicellular tumour spheroids 1204
Multidisciplinary care 427
Multidrug-resistant hepatoma cells 1197
Multimeric YIGSR peptides 1898
Multinuclear platinum complex 1912
Multiple cycles 412
Multivariate analysis 1809
Mutations 1565, 1652
Mutation analysis 1571
Myelotoxicity 1058
Keyword index 2051
British Journal of Cancer (1999) 80(12), 2048-2052
(c) 1999 Cancer Research Campaign
Nasopharyngeal carcinoma 1962
Natural compounds 971
Neck irradiation 808
Neomycin 1132
Neopterin 407
Neovascularization 827
Nerve-sparing RPLND 249
Nested case-control studies 598
Neuroblastomas 1190
Neuropeptides 1026
Neutrophils 331
New telomerase assay 1332
NF-k 954
Nitric oxide 1630, 1945
Nitric oxide synthase 1945
Nolatrexed cytotoxicity 1738
Non-Hodgkin lymphoma 1476
Non-small-cell lung cancers 815, 1289
Normal tissue damage 744
Nuclear expression 1046
Nuclear factor-k 954
Nuclear grades 483
Nucleoside transport inhibition 1738
Nucleotide excision repairs 1245
Occupationally derived lung cancer 1987
Oesophageal adenocarcinoma 1652
Oesophageal cancer 32, 387, 1052, 1281,
1366
Oesophageal carcinoma 834, 1617
Oesophageal squamous cell carcinoma 256
Oesophagogastric cancer 269
Oestradiol 1470
Oestrogen 1092, 1271, 2025
Oestrogen receptors 1150, 1453
Oestrogen receptor- 1982
Oestrogen receptor- 2025
Oils 614
OK-432 775
Oncofetal antigens 569
Oncogenes 526, 676
Optical dosimetry 344
Oral cancer 79, 448, 614, 1380
Organisation of services 1958
Ovarian cancer 70, 315, 444, 685, 946,
1236, 1492, 1644, 1665, 1803, 1826 (SC),
1875, 1920
OVCAR 3 946
Overall survival 1453
Over-expression 914
Oxidants 25
Oxidative DNA damage 940
Oxygen concentrations 1518
Oxygen consumption rates 104
P2XM 1185
p15 458
p16 9
p16 458
p16INK4
1175
p16INK4a
670
p16INK4a
gene 1262
p16/MTS1 79
P16/RB pathway 1289
p19ARF
9
p21 1236
p21WAF1
705, 1144
p21WAF1/Cip1
51, 2001
p21WAF1/CIP1
immunohistochemistry 546
9p21-22 1920
p27KIP1
1427
1p36 1623
p37Kip1
51
p43 874
p53 38, 453, 536, 579, 693, 1062, 1069,
1175, 1185, 1289, 1350, 1400, 1658, 1689
p53 1445, 1623, 1968
p53 codon 72 polymorphism 1828 (SC)
p53 gene 59, 1080
p53 mutations 1912
p53 proteins 1075, 1987
p73 1069, 1623
Paclitaxel 815, 1052
PAIs 17
PAI-1 167, 419
Pain 1512
Palliation 1595
Pancreas 935
Pancreatic cancer 438, 1075, 1595, 1754,
1797
Pancreatic neoplasms 1830
Papillomavirus 2008
Papillomavirus, human 621, 1306
Parotid carcinoma 1296
PARP 1035
PARP cleavage 1892
-Particle emitters 175
Pathology reporting 563
Patient participation 242
Patterns of care 1275
PCNA 387
32
P-Colloid 1955
PCR-SSCP 1652
PD 098059 1412
PDT 133, 185, 676, 744, 998, 1533
Pentacyclic triterpenes 756
Pericardial effusion 1955
Peutz-Jeghers locus 70
PgR 579
pH 1892
Phage displays 1214
Pharmacokinetics 1786
Pharyngeal cancer 614
Phase I studies 1786
Phase II studies 438
Phosphatase activities 1875
Phosphor images 922
Phosphorus 31 magnetic resonance
spectroscopy 133
Photodynamic therapies 133, 185, 676, 744,
998, 1533
Photofrin 744
Photosensitization 946
Photosensitizers 344
Phyto-oestrogens 1150
Picibanil 775
PIFs 1231, 1734
Pituitary tumours 44
Plasma 495, 1995
Plasma DNA 1262
Plasminogen activator inhibitors 17
Plasminogen activator inhibitors type 1 419,
167
Pleural mesothelioma 1781
31
P-MRS 133
Polarized cells 1012
Poly(ADP-ribose) polymerase 1035
Poly(ADP-ribose) polymerase cleavage 1892
Polyethylene glycol 1533
Polymerase chain reaction 67
Polymorphic amino acid 1405
Polyvinyl alcohol 1533
Poor prognosis 483, 843
Population-based audits 609, 1958
Porphobilinogen deaminase 998
Porphyrin 1332
Post-menopausal women 1852
pRB 1875
Predictive values 1599
Prednisolone 412
Pregnancy 1092
Prepuberty 1682
Preterm infants 609
Primary chemotherapy 519
Primary tumour characteristics 1974
Progelatinase A 1137
Progesterone 1271
Progesterone receptors 379, 1453
Progestin 1092
Prognosis 301, 419, 477, 546, 918, 1069,
1648, 1658, 1809, 1968
Prognostic factors 1296
Prognostic impact 286
Proliferation 546, 1062, 1400, 1635
Promoters 1935
Propylnitrosourea 855
Prospective studies 930
Prostate cancer 371, 591, 657, 930, 1565
Prostate cancer pathology 546
Prostate neoplasms 477, 1107
Prostatic carcinomas 309, 1512
Protein kinase C 1558
Protein stability 657
Protein truncation tests 1979
Protein tyrosine kinase 954
Proteins 1107
Proteolysis-inducing factors 1231, 1734, 1884
Protoporphyrin 676
Protoporphyrin IX 185, 998
Psychiatric morbidity 766
Psychological interventions 1770
Psychotherapy 1770
PTEN 9, 904
PTTs 1979
Pulsed-lasers 344
Pyridinoline cross-links 1265
11q24.1-q25 843
16q 556
QTL analysis 855
Quality of life 262, 766, 1588
Quality measurements 563
Quantification 922
Quantitative RT-PCR 883
Quercetin 1150
Question prompt sheets 242
Radiation risks 1461
Radiation therapies 236, 387, 693
Radioiodinated hCG 1582
Radioluminography 922
Radiosensitivity 1400
Radiotherapy 142, 1296, 1588, 1599, 1792
Randomized trials 262
Rats 161, 855, 1512, 1905
rck/p54 914
Reactive oxygen intermediates 371
Reactive oxygen species 25
Receptors 1271
Receptor activation 650
2052 Keyword index
British Journal of Cancer (1999) 80(12), 2048-2052 (c) 1999 Cancer Research Campaign
Recombinant immunotoxins 1214
Recombinant inbred strains 855
Recurrence 448
Ref-1 940
Referrals 427
Registries 1440
Relapses 1392
Renal cancers 1312, 1565
Renal cell cancers 301, 1648, 2001
Reproductive factors 609
RER+ tumours 11
Resistance 1342, 1405
Response rates 1955
RET 383
Retinoblastoma 79
Retinoblastoma protein 670, 705, 1175
Retinoic acid 935, 1494
Retinoic acid receptors 206
Retinoids 1, 1350
Retinoid x receptors 206
Retrotransposon 1312
Reverse transcription polymerase chain
reaction 2019
Review 930
Rhabdomyosarcoma 918
Ribozymes 1558
Risk 1644
Risk factors 621, 1844, 1859
Rodents 724
RPLND 801
RT-PCR 1920
RT/PCR of fine needle aspiration material
73, 519, 1672
Rural areas 1275
S100A4 127
Safflower oil 1132
Sarcomas 1565
Sarcoma botryoides 403
SCID mice 1898
Screening 1644
Seminoma 1859
Sera 1630, 1803, 1987
Serum anti-p53 antibodies 483
Serum CEA 1373
Sex hormone-binding globulins 1470
Sexual functioning 801
Shear flow 1927
Sialic acid surface binding 1754
Sialyl-Lewisx
1169
sIL-2R 407
Singlet oxygen 360
Skin cancer 1087, 1501
Smad 194
Small intestine 1440
Small-cell lung cancer 1026, 1935
Small-cell lung cancer anaemia 396
Sodium butyrate 705, 1617
Soft tissue sarcoma 1185, 1350
Solid tumour genetics 862
Soluble adhesion molecules 273
Soluble CD44 1995
Soluble CD95 (Fas/APO-1) 1648
Somatostatin analogues 1197
S-phase fraction 419
Sphingomyelin pathways 693
Spinal cord 1512
Squamous carcinoma cells, human 1137
Squamous cell carcinoma 1087
Squamous cell carcinoma, early stage central
type 1435
Staging 444, 1259
stat1 1236
Steroid hormones 930
Strand breaks 981
Sulphated glycoprotein 1734
Sulphated glycosaminoglycans 1359
Superoxide dismutase 25
Surgeons 1492
Surgery 444, 1296
Survival 786, 821, 1259, 1392, 1958
Survival analysis 536, 1427
Susceptibility 1405, 1838
Swainsonine 87, 1754
Synergism 786
Synovial sarcoma 1809
Systematic reviews 1275
TAP 639
Targeting 922
Taxoids 1162
Taxol 1020
T-cells 874
Telomerase 1156
Telomerase activities 453
Telomerase inhibitors 1332
Telomere length 1332
Tenascin 685
Testicular cancer 249, 801, 1098, 1571,
1859
Testicular neoplasms 1577
Testicular teratoma 149
Testosterone 2025
TGF- 1708
TGF- 194, 685, 1144, 1708
TGF- receptors 194
Thalidomide 716
Therapies 175
Thiocoraline 971
Thymidine phosphorylase 1726
Thymidine rescue 1738
Thyroid cancer 650, 1461
Thyroid disease 808
TIMP-1 495, 504
TIMP-2 315
Tirapazamine 1245
Tissue-type plasminogen activators 286
T-lymphocytes 229
T-lymphoma 855
TNF 161, 716
TNF- 331
Tobacco smoke 1445
Topoisomerase I 364, 1786
Topoisomerase II 1786
Topotecan 1380
Townsend score 604
Toxicity 1767
tPA:PAI-1 complex 286
Transcription 1935
Transfection 1169
Transitional cell carcinoma 489, 904
TRT-PCR 1672
Tubulin 1020
-Tubulin isotypes 1162
Tumorigenicity 1115
Tumours 117, 344, 724
Tumours, human 1635
Tumour barriers 964
Tumour biology 419
Tumour blood flow 1945
Tumour development 1501
Tumour growth 1945
Tumour immunity 1420
Tumour immunology 1501
Tumour invasion 1708
Tumour markers 221
Tumour metastasis 1898
Tumour necrosis factor 161, 716
Tumour progression 379, 504
Tumour regression 1501
Tumour suppressors 670
Tumour suppressor genes 489, 526, 850,
1623
Tumour targeting 1718
Tumour-specific arginine vasopressin
promoter activation 1935
Tumour-stromal interactions 1708
Turkey 1303
Twins 1098
Tyrosinase 883
Tyrosinase reverse transcription polymerase
chain reaction (RT-PCR) 1672
UFNAC 1672
Ultrasound guided fine needle aspiration
cytology (FNAC) 1672
Ultrasounds 1644
uPA 167, 419
u-PAR 1884
Up-regulation 1617
Urine 273
Urokinase 17, 1884
Urokinase plasmingen activators 536
Urokinase-type plasminogen activators 167,
419
Uruguay 591
Uterine cervix 51
Uterine leiomyosarcoma 1658
Vaccinations 1420, 1483
Variant mRNA 379
Vascular endothelial growth factor 827, 898,
1506
Vascular endothelial growth factor-C 309
Vascular patterns 724
Vascular permeability 1945
Vascular targeting 175
Vasopressin 1935
Vegans 1470
Vegetarians 1470
VEGF 104, 142, 892, 1366, 1518, 1630,
1697
VEGF receptor-3 309
Vesnarinone 96
Viral aetiology 1103
Viral integration 2008
Vision disorders 1459
Volume of work 1958
Vulvar carcinoma 38
WAF1 1445
Weight gain 1852
Wheatgerm agglutinin 1754
WR-2721 629
Xenografts 96, 352, 724
YAG-optical parametric oscillator lasers
(YAG-OPO lasers) 133
Young age 843
Zearalenone 1682
Zingiberaceae 110

				

